# A Cross-Comparison of High-Throughput Platforms for Circulating MicroRNA Quantification, Agreement in Risk Classification, and Biomarker Discovery in Non-Small Cell Lung Cancer

**DOI:** 10.3389/fonc.2022.911613

**Published:** 2022-07-19

**Authors:** Chiara Gargiuli, Loris De Cecco, Andrea Mariancini, Maria Federica Iannò, Arianna Micali, Elisa Mancinelli, Mattia Boeri, Gabriella Sozzi, Matteo Dugo, Marialuisa Sensi

**Affiliations:** ^1^ Platform of Integrated Biology Unit, Department of Applied Research and Technology Development, Fondazione IRCCS Istituto Nazionale dei Tumori, Milan, Italy; ^2^ Tumor Genomics Unit, Department of Research, Fondazione IRCCS Istituto Nazionale dei Tumori, Milan, Italy

**Keywords:** liquid biopsy, circulating microRNAs, high-throughput platforms, lung cancer, microRNA signature classifier, miR-150-5p, miR-210-3p, profiling

## Abstract

**Background:**

Circulating microRNAs (ct-miRs) are promising cancer biomarkers. This study focuses on platform comparison to assess performance variability, agreement in the assignment of a miR signature classifier (MSC), and concordance for the identification of cancer-associated miRs in plasma samples from non‐small cell lung cancer (NSCLC) patients.

**Methods:**

A plasma cohort of 10 NSCLC patients and 10 healthy donors matched for clinical features and MSC risk level was profiled for miR expression using two sequencing-based and three quantitative reverse transcription PCR (qPCR)-based platforms. Intra- and inter-platform variations were examined by correlation and concordance analysis. The MSC risk levels were compared with those estimated using a reference method. Differentially expressed ct-miRs were identified among NSCLC patients and donors, and the diagnostic value of those dysregulated in patients was assessed by receiver operating characteristic curve analysis. The downregulation of miR-150-5p was verified by qPCR. The Cancer Genome Atlas (TCGA) lung carcinoma dataset was used for validation at the tissue level.

**Results:**

The intra-platform reproducibility was consistent, whereas the highest values of inter-platform correlations were among qPCR-based platforms. MSC classification concordance was >80% for four platforms. The dysregulation and discriminatory power of miR-150-5p and miR-210-3p were documented. Both were significantly dysregulated also on TCGA tissue-originated profiles from lung cell carcinoma in comparison with normal samples.

**Conclusion:**

Overall, our studies provide a large performance analysis between five different platforms for miR quantification, indicate the solidity of MSC classifier, and identify two noninvasive biomarkers for NSCLC.

## Introduction

MicroRNAs (miRs) are a class of small (18 to 22 nt) non-coding RNAs with known roles in gene regulation (1–3). miRs can be released from cells into the extracellular space and have been detected in all tested biological fluids (1–5). Circulating miRs (ct-miRs) are either stored in particles (exosomes, microvesicles, and apoptotic bodies) or associated with RNA-binding proteins or lipoproteins, which prevent their degradation (3–5). The stability, abundance, and variety of ct-miRs made them attractive candidates as non-invasive biomarkers for diagnosing, predicting, and monitoring diseases like cancer (6–8), and increasing attention is being paid to their role in lung carcinogenesis (9–16).

In our institution, the use of ct-miRs for the early detection of lung cancer has been assessed as a complementary diagnostic tool in the context of low-dose computed tomography (LDCT) screening in large retrospective cohorts (12, 13). These studies led to the development of a plasma miR signature classifier (MSC) based on reciprocal ratios of 24 plasma miRs able to stratify individuals undergoing lung cancer screening into three levels (high, intermediate, and low) according to the risk of developing lethal lung cancer (13, 14). As assessed in samples collected from smokers within the randomized Multicenter Italian Lung Detection trial, a large retrospective validation study, MSC resulted in a sensitivity, specificity, positive predicted value, and negative predictive value of 87, 81, 27, and 99% (13). The utility of the classifier was also recently assessed, thanks to the prospective BioMILD screening trial on 4,119 high-risk volunteers, where MSC-positive participants had a 2-fold higher risk to develop lung cancer within the fourth year of screening than MSC-negative participants, independently of the low-dose computed tomography (LDCT) result (14). The risk level given by MSC reflects microenvironment-related changes associated to lung cancer development and aggressiveness. In detail, the miRs composing the classifier were found to be associated to an immunosuppressive phenotype of specific immune cell subsets, such as neutrophils, macrophages, and lymphocytes (15).

Several high-throughput platforms, based on quantitative reverse transcription PCR (qPCR) or on sequencing (miR-Seq), have been routinely used to quantify miRs in human plasma. However, there is poor consensus on the optimal methodology for the successful clinical application of ct-miR biomarkers (17–21). Pre-analytical and analytical conditions are a major source of variation in results, but many challenges remain in terms of the reliability of ct-miR quantification methods (17–21). In 2014, the “microRNA quality control study” (miRQC) systematically evaluated 12 available miR platforms across a variety of samples including human universal reference RNA, human brain RNA, and human serum samples (17). The expression level of 196 common miRs was considered. Although no platform was consistently superior to the others, there was substantial variability in performance assessments. Only two miRs (3%) were differentially expressed (DE) by all platforms; about half of the miRs (48%) were concordant for half of the platforms. Since the miRQC study, newer platforms have emerged. Nonetheless, most recent studies report similar findings when comparing the different platforms for profiling low-copy number miRs in human biological fluids (plasma/serum) or extracellular vesicles (18–21).

A few reports have compared ct-miR abundance using multiple high-throughput technologies in defined clinical subgroups. Only one study has reported the use of multiple platforms (Toray 3D Gene System from Toray Systems, nCounter from Nanostring Technologies, and QIAseq from Qiagen) to profile cell-free and extracellular-derived miR fractions from non‐small cell lung cancer (NSCLC) patients and healthy donors (20). The patients’ cohort was however heterogenous and not age-matched with the control group, preventing the interpretation of differential expression between NSCLC patients and healthy control samples for different ct-miR fractions and platforms (20). In addition, to the best of our knowledge, none of the previous studies has challenged the ability of different platforms to correctly classify individual samples according to a clinically relevant ct-miR signature.

To address these issues, we determined the miR profile of plasma samples from 10 stage IV NSCLC patients and 10 healthy heavy smokers matched for age, sex, smoking status, and MSC classification assessed with the gold-standard method (13, 14), using five well-established high-throughput methods. Three of them, Taqman OpenArray/Taqman OpenArray Advanced from Thermo Fisher Scientific and miRCURY LNA from Qiagen, were qPCR-based. The remaining two, EdgeSeq from HTG Molecular and QiaSeq miRNA Library from Qiagen, were next generation sequencing (NGS)-based. EdgeSeq allows the assessment of 2,083 human miR transcripts directly from plasma, without extraction, through quantitative nuclease protection, whereas QiaSeq is a true discovery platform enabling the capture of the whole miRNome profile.

The aims of this cross-platform comparison were assessment of intra- and inter-platform reproducibility, agreement in correctly classifying samples according to the MSC classifier, and identification and validation of putative cancer-associated ct-miRs.

## Materials and Methods

### Characteristics of the Participants

Blood was collected from stage IV NSCLC patients and heavy smoker healthy individuals, as controls, with no history of cancer or other diseases. Patients and controls were classified, according to their class of risk, based on the reference MSC test generated from the ratios of 24 plasma miRs (12–14). The test was performed, as previously described, using a Custom RT and Pre‐amplification Pools with TaqMan MiR Assays (Thermo Fisher Scientific, Waltham, MA, USA) (12–14). The clinical characteristics and MSC scores of the participants to the study are listed in **Table 1**. Only individuals belonging to high and low risk were included in the study. There was no significant difference in sex, age, smoking history, and nationality between the participants (*p* > 0.05).

**Table 1:** Clinicopathological features of the analyzed cohort.

Characteristics	Non‐small cell lung cancer (NSCLC) Patients (*N* = 10)	Healthy Controls (*N* = 10)
NSCLC types		
Adenocarcinoma	5	0
Sarcomatoid	2	0
Others^a^	3	0
Stage		
IV	10	0
Gender		
Male	7	7
Female	3	3
Age		
>50	10	10
Smoking status		
Current smokers	10	10
MSC status		
High	5	5
Low	5	5

a Others: adenosquamous (N = 1), poorly differentiated (N = 1), and squamous (N = 1).

### Plasma Preparation and RNA Extraction

Blood samples, collected in P100 tubes (BD Bioscience, San Jose, CA, USA), were separated within 2 h of collection into plasma aliquots by two centrifugations of 1,600*g* for 10 min and stored at -80° until assayed. Total RNA was extracted from 200 μl of plasma using the automatic nucleic acid extractor Maxwell 48 (Promega, Madison, WI, USA), eluted in nuclease-free water, and stored at –80°C. Exogenous synthetic miRs (ath-miR-159a, cel-miR-39-3p, UniSp2, UniSp4, UniSp5, and UniSp6) (Thermo Fisher Scientific and Qiagen, Hilden, Germany) were added as spike-in controls during sample processing to minimize the loss of the specific RNA template and to monitor the extraction efficiency.

### ct-miR Profiling and Quality Controls

The Taqman OpenArray Human microRNA panel (OAC as Open Array “Classic” assay) (Thermo Fisher Scientific) is a fixed-content panel containing validated human TaqMan miR assays derived from Sanger miRBase release v.14. In total, 754 human miRs are amplified in each sample together with 16 replicates each of 4 internal controls (ath-miR159a, RNU48, RNU44, and U6 rRNA). In brief, according to the manufacturer’s instructions, separate reverse transcription (RT) and pre-amplification reactions were performed on all samples using MegaPlex Pools A (v2.1) and B (v3.0) primer pools, which reverse-transcribe and pre-amplify specific miRs. The pre-amplified products were diluted before mixing with TaqMan OpenArray Real-Time PCR Master Mix and loaded onto a 384-well TaqMan OpenArray loading plate.

The Taqman OpenArray Human Advanced MicroRNA Panel (OAA) (Thermo Fisher Scientific) is also a fixed-content panel containing 754 well-characterized human miR sequences from the Sanger miRBase release v.21. The internal controls are ath-miR-159a and cel-miR-39-3p. Preparation of poly(A) tailing and adapter ligation reactions were performed, according to the manufacturer’s instructions, on all samples before RT and set-up of qPCR in a 384-well TaqMan OpenArray loading plate. The OAC and OAA products were automatically loaded from the 384-well plates onto the OpenArray plates using the AccuFill System (Thermo Fisher Scientific), and the qPCR reactions were carried out on a QuantStudio 12K Flex Real Time PCR system (Thermo Fisher Scientific). Quality controls were performed on raw data to control for batch effects and outliers. The distribution of raw Ct/Crt, AmpScore, and CqConf values of the exogenous spike-in ath-miR-159a was evaluated. Plate images were manually inspected for every sample in every run to control for evaporation, bubbles, or oil leakage. The fluorescence of ROX, a passive dye in the qPCR reagent mix, was controlled to confirm that each well was correctly loaded. Wells with a ROX signal above 1,000 were included.

The miRCURY LNA miRNome PCR Panels (miRCURY) (Qiagen) is a system based on universal RT, followed by qPCR amplification with locked nucleic acid (LNA)-enhanced primers designed for miR detection using SYBR tracking dye. In each sample, a total of 752 unique human miRs based on Sanger miRBase release 21 are profiled using miRNA ready-to-use PCR human panels I and II following the manufacturer’s instruction. The PCR panels also include three small RNA reference genes (U6, SNORD38B, and SNORD49A) and three miR reference genes (miR-103-3p, miR-191-5p, and hsa-miR-423-5p), all found on panel I. Panel I also contains qPCR assays for the 5 synthetic RNAs in the RNA Spike-in Kit (cel-miR-39-3p, UniSp2, UniSp4, UniSp5, and UniSp6). After RT, qPCR reactions were carried out on a QuantStudio 12K Flex Real Time PCR system. To control for run-to-run variations, interplate calibration was performed using the six interplate calibrators, UniSp3 miR, as per the manufacturer’s instruction. After the calibration of each plate, the data were merged to obtain a unique data matrix.

The QiaSeq miRNA Library (QiaSeq) (Qiagen) is a discovery platform which captures all small RNA sequences and uses unique molecular indices (UMIs) to enable an unbiased and accurate miRNome-wide quantification of mature miRs by NGS technology. Briefly, the preparation of small RNA libraries was performed according to the manufacturer’s procedures. The quality and concentration of libraries were determined using Qubit™ DNA HS Assay Kit on a Qubit fluorometer (Thermo Fisher Scientific), while the library size was assessed using Agilent High Sensitivity D1000 ScreenTape on a 4200 TapeStation, (Agilent Technologies, Santa Clara, CA, USA). The libraries were sequenced on a NextSeq 500 System (Illumina, San Diego, CA, USA). Raw sequences were analyzed using the Qiagen Online Data Analysis Center with default settings, and 1,823 unique miRs were selected for the subsequent analysis.

In the EdgeSeq miR Whole Transcriptome Assay (EdgeSeq) (HTG Molecular Diagnostics, Inc., Tucson, AZ, USA), frozen plasma samples were shipped to HTG to carry out the multiplexed nuclease protection assay, sequencing, quality controls, and primary analysis of the data. The assay, which allows the assessment of miRs directly, without extraction, is based on probes containing sequences complementary to 2,083 specific miRs (miRBase v20) and flanking sequences for downstream amplification. It includes five negative process control probes to the plant gene: “ANT” (Aintegumenta, NM_119937). Probes that successfully hybridize to their cognate miR in the sample are protected from nuclease digestion, amplified with the addition of barcodes, and then sequenced on automated HTG EdgeSeq sequencer system. This study was executed at HTG Molecular in the VERI/O Laboratory following VERI/O processes and procedures. Data are provided as a data table of raw counts, QC raw, and log_2_CPM (counts per million).

### Data Import and Processing

All statistical and bioinformatic analyses were performed using the R statistical program v. 3.6.1. For the three qPCR-based panels, text files were downloaded from the QuantStudio 12K Flex and were imported in R as data tables. The expression matrices in qPCRset format were created for every dataset using the HTqPCR R package (22). Filtering on detection was performed according to the manufacturers’ suggested thresholds: Crt ≦ 28, AmpScore > 1, and CqConf > 0.8 for OAC and OAA panels; Ct ≦ 35 and AmpScore > 1 and CqConf > 0.8 for miRCURY panels I + II. If miRs did not reach the thresholds, they were set to 40 and considered as “undetected”. Since different miRbase versions were used to design the platforms, we downloaded the platform annotations from each manufacturer’s website and, using the mature sequence identifier, we converted miR names to miRbase version 21. For qPCR-based platforms, data were normalized using the global median normalization method with the median values of detectable miRs. For QiaSeq, primary analysis was performed with the GeneGlobe online software (https://geneglobe.qiagen.com/sg/analyze/). Raw counts were normalized using the trimmed mean of M-value (TMM) method (23) implemented in the edgeR package (24) considering that only the UMI counts had more than 10 counts mapping in at least 30% of samples. For EdgeSeq, raw counts were corrected by background subtraction of the maximum value of the five ANT probes. In addition, control miRs were removed, and miRs with negative counts after the background correction were set to 0 for the subsequent normalization performed using the TMM method (23).

### Guanine-Cytosine Content Evaluation

Guanine–cytosine (GC) content was calculated for detected and undetected miRs common to all platforms (*n* = 488). The percentage of GC was calculated as the sum of G and C present in every miR sequence divided by the length of the sequence and multiplied by 100. Differences between detected and undetected miRs in each platform were assessed with Wilcoxon rank-sum test.

### Correlation and Concordance Analysis

**Figure 1:**
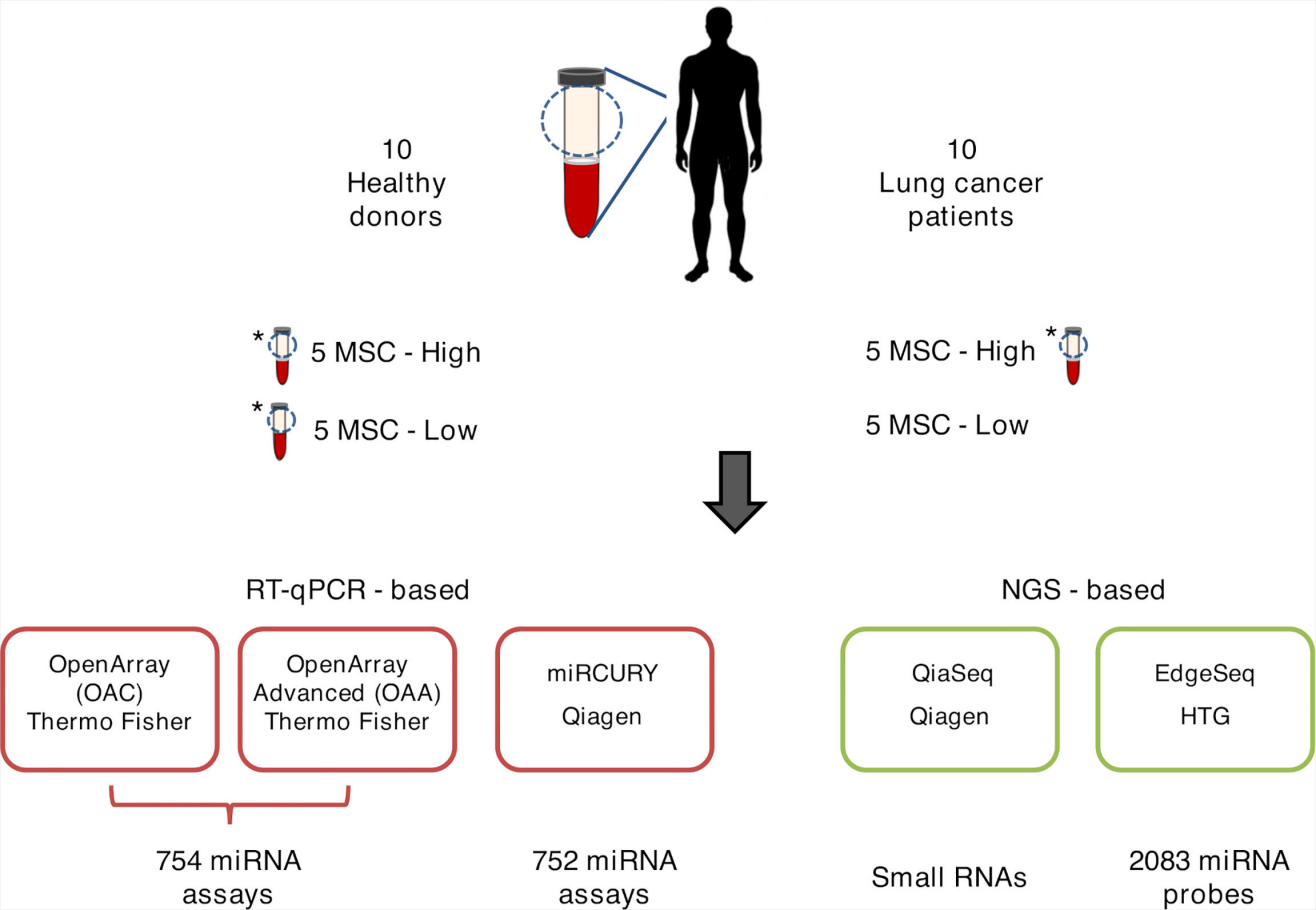
Graphical representation of ct-miR profiling in non‐small cell lung cancer (NSCLC). The plasma samples of ten stage IV NSCLC cancer patients and ten healthy heavy smoker donors were quantified for miR expression by five different high-throughput platforms—three qPCR-based (lower-left panels, boxed in red) and two next-generation sequencing-based (lower-right panels, boxed in green). Three samples were tested in duplicate and are marked with an asterisk.

Three samples deriving from a patient and two healthy subjects were profiled twice each using, depending on the platform, ether independent RNA extractions of the same plasma or duplicate aliquots of crude plasma (**Figure 1**). The concordance and correlation coefficient (CCC) was calculated using the ΔCt/Crt and log_2_(CPM) values on pairs of technical replicates for each platform with the epi.ccc function of epiR package (https://cran.r-project.org/web/packages/epiR/epiR.pdf). Hierarchical clustering was performed using Euclidean distance and Ward method.

### MSC Algorithm

The plasma-based MSC test analyzes the reciprocal levels of 24 ct-miRs (listed in **Supplementary Table S1**) by qPCR. The expression values of these miRs were determined by gold-standard methodology. Briefly, the Multiplex Pools Protocol on custom-made microfluidic cards (Thermo Fisher Scientific) containing the 24 miRs spotted on duplicates was used as described (14, 15). To remove the batch effect, a ratio-based approach, using the gold-standard methodology as reference array, was first adopted (25). In detail, the normalized data of the 24 miRs from each platform were scaled by the arithmetic mean of the reference array. The fixed MSC algorithm (26) was then applied to the 24 scaled miR profile obtained for each sample in each platform, taking into account the single values. The MSC risk scores were compared with those calculated in the same samples by the gold-standard methodology (**Table 1**). Cohen’s kappa was used to assess the agreement between platforms for MSC classes.

### Differential Expression Analysis and Concordance Rate Between Platforms

Differential expression analysis was carried out on normalized data using the linear modeling approach implemented in the limma package (27). Nominal *p*-values were corrected for multiple testing using the Benjamini–Hochberg false discovery rate (FDR). DE ct-miRs were selected according to an FDR <0.1 in all the platforms. We then assessed the pairwise concordance of fold changes (FC) between platforms (platform X vs. platform Y). Four qualitative evaluations were assigned to each comparison: compressed, opposite, overestimate, or concordant (28). When the compared FC were in the same direction but the ratio of X/Y was greater than or equal to 2, a value of “compressed” was assigned. Similarly, if the FC ratio of X/Y is less than or equal to 0.5, the comparison was deemed “overestimate”. FC ratios between these values were named “concordant”. When two FC values were not in the same direction and either of them was greater than 2 or less than 0.5, the comparison was determined to be “opposite”. Concordance rates were calculated by number of miRs with “concordant” and “overestimate” calls divided by the total number of analyzed miRs which were in common and expressed in all the platforms.

### Individual qPCR Assays

Single qPCR reactions were performed using TaqMan MicroRNA Assays (hsa-miR-150-5p and hsa-miR-93-5p, Thermo Fisher Scientific) according to the manufacturer’s instructions. Briefly, total RNA (3 µl) was reverse-transcribed, and the resulting cDNA was used (2.5 µl) for the pre-amplification reaction. The pre-amplified cDNA was diluted 1:12, and 0.10 µl of the product was used to perform the qPCR amplification reaction using the corresponding miR assay primers and TaqMan Universal PCR Master Mix no AmpErase UNG, according to the manufacturer’s instructions. The PCR reaction conditions were as follows: enzyme activation at 95°C for 10 min, 40 cycles of denaturation at 95°C for 15 s, and annealing/extension at 60°C for 60 s. The amplification was performed in 384-well plates with QuantStudio 12K Flex Real Time PCR system (Thermo Fisher Scientific) assembled using the Janus automated workstation (PerkinElmer, Waltham, MA) from 96-well plates. Each qPCR analysis was done in triplicate, and data were acquired through QuantStudio 12K Flex v.1.2.3; the obtained mean Ct values were exported for statistical analysis. miR-93-5p was identified as a reference housekeeper by all the platforms using the selectHKgenes function with Vandesompele method (29) of SLqPCR R package (https://bioconductor.org/packages/release/bioc/html/SLqPCR.html) calculated on filtered raw data of each platform. The expression levels of miR-150-5p were then normalized according to the DCt method (30) using the Ct mean values of the endogenous control.

### External Validation

External validation was performed in The Cancer Genome Atlas (TCGA) dataset. Raw count values for the TCGA miR-seq data of lung adenocarcinoma (LUAD) and lung squamous cell carcinoma (LUSC) were downloaded from the Genomic Data Commons data portal (https://portal.gdc.cancer.gov/). The LUAD project included 519 primary solid tumors, 2 recurrent tumors, and 46 normal samples from adjacent tumor tissues; the LUSC project included 478 primary solid tumors and 45 normal samples (31, 32). TCGA raw count values, samples, and patients’ annotations were obtained using the TCGABiolinks package (33). miRs with less than 10 counts expressed in more than 50% of samples were filtered out. Raw counts were then normalized with the TMM method implemented in the edgeR package (23, 24). Differential expression between tumor and normal tissue was performed using the limma/voom method (27). Nominal *p*-values were corrected for multiple testing using the Benjamini–Hochberg FDR.

### ROC Curves

Receiver operating curves (ROC) with area under the curve (AUC) calculation were used to determine the diagnostic value of miRs in distinguishing between plasma from healthy controls and NSCLC patients (34). ROC curves were obtained by plotting sensitivity against specificity using the ROC function of pROC R package (34). An area greater than 0.5 under the curve suggests the diagnostic potential of each ct-miR candidate.

## Results

### Study Design and ct-miR Expression Profiling

**Figure 2:**
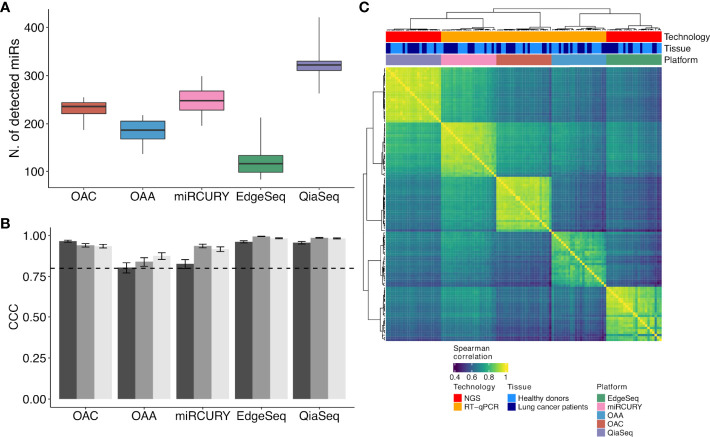
ct-miR detection and correlation and concordance analysis across duplicates and platforms. **(A)** Boxplots representing the number of detected ct-miRs in each platform, calculated after normalization and filtering, with respect to the 488 common miRs. **(B)** Grouped bar plots showing the concordance and correlation coefficient calculated among the three duplicates in each platform for the 488 common miRs. The vertical bars indicate the 95% confidence interval of the correlation. The horizontal dotted line represents the threshold of the minimum correlation value, 0.8. The black, gray, and light gray bars refer to technical duplicates from three plasma samples. **(C)** Correlation heat map showing the agreement between the five platforms. Spearman correlation was calculated on the samples’ z-scores of each platform considering the 488 miRs common to all platforms. Hierarchical clustering was performed using Euclidean distance and Ward linkage.

A total of 20 human specimens were employed for this study, which included plasma from NSCLC patients (*n* = 10) and healthy subjects (*n* = 10) matched for age, sex, smoking status, and MSC risk score (**Table 1**). Three plasma, one derived from a patient and two from healthy subjects, were in duplicate, bringing the total number of analyzed samples to 23 (**Figure 1**). RNA derived from these samples was profiled by the following four high-throughput technological platforms: Taqman OpenArray Human miR and Taqman OpenArray Human Advanced miR Panels (Thermo Fisher Scientific), miRCURY LNA miR miRNome PCR Panels (Qiagen), and QiaSeq miRNA Library (Qiagen) (**Figure 1**). The fifth platform, EdgeSeq miR Whole Transcriptome Assay (HTG Molecular Diagnostics), employed crude blood plasma instead (**Figure 1**). The starting material for the duplicates was a second aliquot of either crude plasma (for EdgeSeq platform) or RNA independently extracted (for all remaining platforms). Their inclusion was required to assess intra-platform repeatability as described below. The presence and detection of miRs by platform and sample is reported in **Supplementary Table S2**. The number of common miRs detectable by all platforms was 488 (**Supplementary Figure S1A** and **Supplementary Table S2**). For each platform, the average number of ct-miRs detected after normalization and filtering in the different samples ranged from 236 for EdgeSeq platform to 806 for QiaSeq (**Supplementary Figure S1B**). By considering only the 488 commonly detected miRs, average detection ranged from 120 to 323 (**Figure 2A**). As shown in **Supplementary Figure S1C**, the influence of GC content had no or little impact on the detection rate. The 488 common miRs included a list of 26 miRs (named super_core in **Supplementary Table S2**) highly expressed in all plasma samples as indicated by the empirical cumulative distribution curves of their expression quantiles (**Supplementary Figure S1D**).

### Intra-platform Repeatability and Inter-platform Comparison

**Figure 3:**
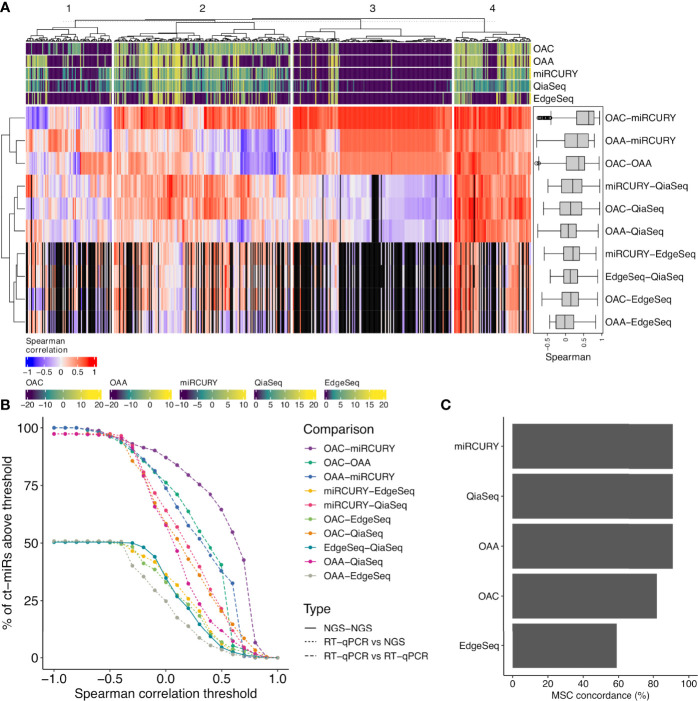
Correlation analysis between each pair of platform and concordance assessment for the miR signature classifier (MSC). **(A)** The correlation heat map shows how the different platforms correlate with respect to the expression values of the 488 common miRs. The median pairwise Spearman’s correlation values are shown also as boxplots in a black box (right corner). The colored bars on the top and bottom of the heat map (violet- to yellow-colored gradient) define the median-normalized expression values of each platform. Four functional groups are identified and defined according to different levels of correlation (1, scarce; 2, intermediate; 3, both positive (red) and negative correlation (blue); 4, high). The black vertical bars represent the miRs with an expression value of 0 in EdgeSeq platform that do not correlate. **(B)** Curves showing the number of ct-miRs correlated above increasing correlation cutoffs for each pairwise comparison between platforms. **(C)** Bar plots displaying the percentage of concordance in assigning the label miR risk classifier MSC—high or MSC—low compared with the reference platform (Custom-made Microfluidic Cards, Thermo Fisher) used to calculate the clinical validated score.

To evaluate the intra-platform repeatability, we calculated for each platform the Lin’s CCC between the ct-miR profiles of duplicate samples. CCC between duplicates was >0.8 for all platforms considering either the 488 common miRs (**Figure 2B**) or the total number of available miRs (**Supplementary Figure S2**). Pairwise scatterplots for duplicates are displayed in **Supplementary Figure S2**. These results demonstrate intra-platform consistency and no significant differences among the different technologies. We then calculated Spearman’s correlation coefficients between pairs of samples within and between platforms. Hierarchical clustering of the correlation matrices showed that each platform produced very homogenous and highly correlated data (**Figure 2C**). Within each platform, we did not observe any separate cluster of tumor and normal samples. This suggests that, independently of the platform, most of the ct-miRs are uninformative to distinguish the two groups. We did instead observe clustering according to the profiling platform, indicating that the variability explained by the technological approach is higher than the biological variability. An unsupervised hierarchical clustering algorithm was carried out on Spearman’s correlation coefficients calculated between the pair of platforms for each of the 488 common miRs. Four major clusters were identified according to different levels of correlation (**Figure 3A**). Cluster 1 comprised 17% of miRs displaying the lowest inter-platform correlation for all pairs of platforms. Cluster 3 included 32% of miRs that had low expression levels in all platforms and that were highly correlated when comparing qPCR-based platforms but were negatively correlated between NGS- and qPCR-based platforms. Cluster 2 included 35% of miRs and showed a heterogenous pattern of correlation. A first subset of miRs showed a positive correlation in all comparisons, whereas a second subset showed negative correlations when the comparisons were against OAA, indicating that the expression of these ct-miRs is inconsistent specifically for this platform. Finally, cluster 4 included 15% of miRs that were highly expressed in all platforms and showed a high inter-platform correlation. EdgeSeq did not correlate with any other platforms since many of the 488 miRs showed an expression value of 0 in all samples. miRs belonging to each cluster are reported in **Supplementary Table S3**. The Spearman correlation coefficients among the six platforms shown in the right boxplot of **Figure 3A** indicate that the highest inter-platform reproducibility was observed between qPCR-based platforms (miRCURY, OAC, and OAA). For each pair of comparisons between platforms, we counted the number of ct-miRs above increasing correlation cutoffs (**Figure 3B**). We confirmed that, independently of the correlation cutoff, the comparison between qPCR-based platforms returned the highest number of correlated ct-miRs, especially for OAC vs. miRCURY. Comparisons including EdgeSeq showed the lowest number of correlated ct-miRs due to the lower detection rate of EdgeSeq compared with the other platforms.

### Cross-Platform Concordance in the Assignment of a Clinical Validated miR Risk Score

Our cohort consisted of subjects equally distributed within high and low risk (**Table 1**) in both classes (NSCLC and controls) as previously assessed by the gold-standard methodology. MSC algorithm was adopted to classify each sample according to the expression profiles of the 24 ct-miRs (**Supplementary Table S1**) determined in each platform. The classification of each sample (including duplicates from two subjects) for each platform is displayed in **Supplementary Table S4A**. All qPCR-based platforms and QiaSeq displayed a classification highly concordant to the original assessment by the gold-standard method (**Figure 3C** and **Supplementary Table S4A**). The same results were obtained by computing Cohen’s kappa statistics as pairwise measure of similarity when each platform was confronted to the reference (**Supplementary Table S4B**). A lower fidelity was displayed by EdgeSeq when compared with the reference (**Supplementary Tables S4A, B**). Except for EdgeSeq, all other platforms correctly classified all samples from MSC-low individuals, whereas the situation was more heterogeneous for MSC-high individuals **(** **Supplementary Table S4A**). Overall, OAA, miRCURY, and QiaSeq were the three platforms with 91% of correctly classified samples, followed by OAC (82%) and EdgeSeq (59%) (**Figure 3C** and **Supplementary Table S4A**). These results demonstrate that the classification obtained by the standard protocol could be replicated with a good agreement using at least two qPCR-based technologies and one sequencing technology.

### Differential ct-miR Modulation in NSCLC Patients Compared to the Healthy Control Group

To evaluate the differential expression concordance among platforms, we identified DE ct-miRs between NSCLC patients and healthy donors for each platform. The number of miRs that passed the detection filter and were available for the contrast differed among platforms: 689 for QiaSeq, 337 for miRCURY, 305 for OAC, 269 for OAA, and 246 for EdgeSeq. Among the 488 miRs measured by all platforms, those commonly detected were over 80% for qPCR technologies, were 50% for EdgeSeq, and dropped to 44% for QiaSeq. In total, 100 miRs were altogether detected by all platforms, 164 by all but EdgeSeq, which presented the lowest number of ct-miRs passing the detection filters. The results of the DE analysis for all platforms are presented in **Supplementary Table S5**. For each platform, we evaluated the number of DE ct-miRs at varying FDR thresholds, ranging from 0.25 to 0.01 (**Supplementary Figure S3A**). On average, the miRCURY platform gave the highest number of DE ct-miRs, followed by OAC, QiaSeq, and EdgeSeq. No DE ct-miRs were identified for OAA at any FDR threshold. At the usual FDR <0.05, the miRCURY platform gave 43 DE ct-miRs, followed by QiaSeq (*n* = 5) and EdgeSeq (*n* = 1). No DE ct-miRs were found for OAC and OAA at an FDR <0.05. Considering a stringent FDR of 0.01, only QiaSeq identified two DE ct-miRs. We next evaluated the intersection between the lists of DE ct-miRs identified for each platform at different FDR thresholds (**Supplementary Figure S3B**). At FDR <0.01, no DE ct-miRs were shared between two or more platforms. At FDR <0.05, one ct-miR was identified by three platforms and two by two platforms. At increasing FDR, the number of shared DE ct-miRs across platforms increased. Since the selection of DE ct-miRs by different FDR cutoffs influences the comparison of the platforms, we evaluated the correlation of the t-statistics to assess whether at least the direction of the modulation was concordant across platforms (**Supplementary Figure S3C**). All pairwise comparisons between platforms showed positive correlation values, indicating that, on average, the trend of modulation of ct-miRs between lung cancer patients and healthy donors was similar between platforms. However, only OAC vs. miRCURY and QiaSeq vs. miRCURY had correlation values higher than 0.5.

**Figure 4:**
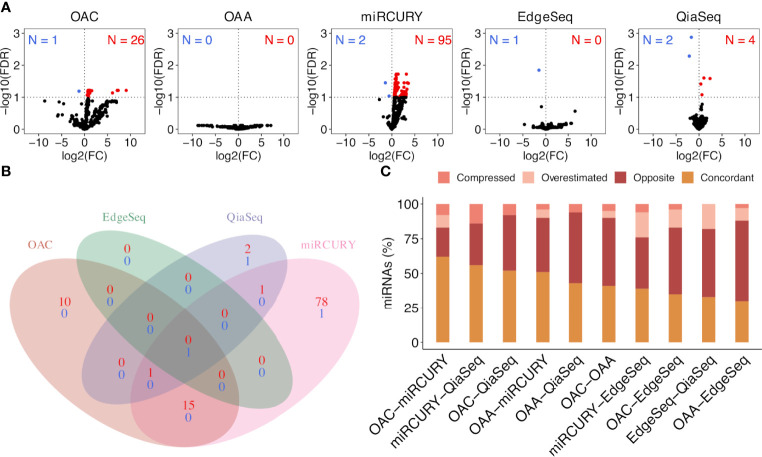
Significantly dysregulated ct-miRs in non‐small cell lung cancer (NSCLC) patients compared with healthy donors and fold change concordance evaluation. **(A)** Volcano plots showing DE ct-miRs between lung cancer patients and healthy donors. The x-axis shows the log2 fold change. The y-axis shows the –log10 of the false discovery rate. A false discovery rate of <0.1, represented by a horizontal dashed line, is used to select DE ct-miRs. The up- and downregulated ct-miRs in lung cancer patients are highlighted in red and blue, respectively. **(B)** Venn diagram reporting the intersection of the ct-miRs significantly upregulated (red) and downregulated (blue) in lung cancer patients across the platforms. **(C)** Stacked bar plots showing the concordance in fold changes between platform pairs expressed in percentage of miRs. The four indices—”compressed”, “opposite”, “overestimated”, and “concordant”—are described in “*Materials and Methods*”.

To select ct-miRs DE in at least four platforms, we therefore applied an FDR cutoff of 0.1. Volcano plots representing the results of the DE analysis between lung cancer patients and healthy controls at an FDR <0.1 are shown in **Figure 4A**. At a threshold of FDR <0.1, we detected 27 DE ct-miRs on OAC, 6 on QiaSeq, 97 on miRCURY, 1 on EdgeSeq, and none on OAA, corresponding to 4.3, 0.5, 5.2, 0.8, 0.4, and 0% of miRs available for the contrast. A Venn diagram displaying the intersection between the lists of significantly up- or downregulated ct-miRs in each platform is shown in **Figure 4B**. Among upregulated ct-miRs, at FDR <0.1, one was common to OAC, miRCURY, and QiaSeq platforms, whereas 16 were commonly detected on two of them. The downregulated ct-miRs included 1 miR shared by miRCURY and QiaSeq and 1 common to the four platforms (miR-150-5p, FDR <0.05 in miRCURY, QiaSeq, and EdgeSeq; FDR <0.1 in OAC).

### Fidelity of Fold Change Across Platforms and Experimental Validation of miR-150-5p

**Figure 5:**
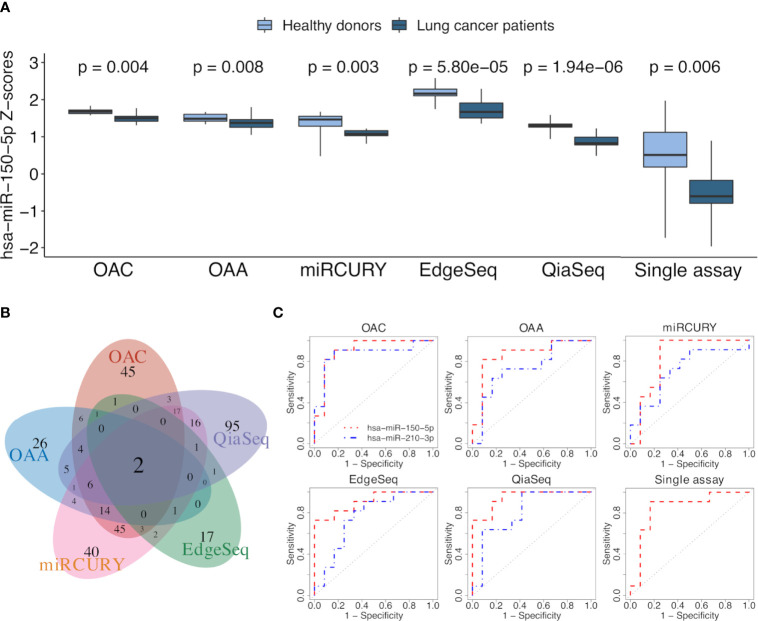
ct-miR differential expression and validation. **(A)** Boxplots reporting the differences in the expression values of miR-150-5p between non-small cell lung cancer (NSCLC) patients and healthy donors in all platforms. Single-assay validation test of miR-150-5p after normalization to the reference miR-93-5p is reported. All *p*-values were obtained using limma, except for the single assay where unpaired two-tailed *t*-test was applied. **(B)** Venn diagram showing the intersection of AUC values above 0.7, calculated on normalized miR values of all the platforms. **(C)** Receiver operating characteristic (ROC) curves of miR-150-5p (orange) and miR-210-3p (blue) obtained by comparing the two groups of lung cancer patients and healthy donors. The area under the ROC curve is above 0.7 for both miRs in all the platforms and even for miR-150-5p in the validation single assay.

We selected all ct-miRs identified as DE in at least one platform and evaluated the fold change concordance between platforms as defined in the “Materials and Methods” section. As shown in **Figure 4C**, the highest rate of concordant miRs was found between OAC and miRCURY, followed by either OAC or miRCURY compared with QiaSeq. The percentage of miRs displaying fold changes in the opposite direction increased when the comparisons were done against EdgeSeq and OAA. miR-142-3p was the concordant upregulated ct-miR across OAC (FDR <0.1), miRCURY (FDR <0.05) and QiaSeq (FDR <0.1) in the plasma of NSCLC patients compared with healthy subjects. The ct-miRs concordantly and significantly upregulated in at least two platforms were as follows: miR-590-3p, miR-766-3p, miR-103a-3p, miR-98-5p miR-296-5p, miR-191-5p, and miR-24-3p (OAC and miRCURY) and let-7f-5p (QiaSeq and miRCURY). Except for miR-142- 3p and miR-98-5p, absent from EdgeSeq, all others belonged to the 488 miRs present on all platforms. The majority was included in cluster 2, whereas miR-590-3p and miR-191-5p were in cluster 4 (**Figure 3A** and **Supplementary Table S3**). The only ct-miR downregulated in cancer patients compared with donors, miR-150-5p (cluster 4, **Figure 3A** and **Supplementary Table S3**), was significantly DE in four platforms (**Figure 5A**). A trend toward significance (nominal *p*-value = 0.008) was also observed in the fifth platform (OAA) (**Figure 5A**). To further investigate the robustness of differences in the abundance of miR-150-5p, individual qPCR assays were performed. By ranking the 26 ct-miRs detected in all samples and platforms according to their average rank across platforms (**Supplementary Table S6**), it was shown that hsa-miR-93-5p is the most stable ct-miR in the cohort and was selected as the normalizer for the single assay. The results confirmed that the relative normalized expression of miR-150-5p in the plasma of NSCLC patients was significantly lower than in healthy donors (**Figure 5A**).

### Discrimination of NSCLC Patients and Controls by Receiver Operating Characteristic Curves

To assess the translation of differential expression into diagnostic power, we evaluated the ability of ct-miRs to discriminate NSCLC patients from controls using a ROC curve analysis. Overall, OAA and OAC showed the highest number of potentially diagnostic ct-miRs, followed by QiaSeq, miRCURY, and EdgeSeq (**Supplementary Figure S4A**). The performance of the platforms in identifying diagnostic ct-miRs varied according to the AUC cutoff selected but, in general, decreased rapidly at increasing values of AUC. At AUC >0.8, miRCURY was the best-performing platform, followed by OAC, QiaSeq, OAA, and EdgeSeq. At AUC >0.9, miRCURY was again the top-ranking platform with 7 diagnostic ct-miRs, followed by QiaSeq, OAC, EdgeSeq, and OAA. We next compared the lists of ct-miRs with AUC above a certain threshold (**Supplementary Figure S4B**). For AUC >0.8, only one ct-miR was shared by at least four platforms. Upon increasing the AUC to 0.9, no shared ct-miRs were found for four and five platforms and only one for at least two or three platforms. The correlation of AUC values showed a poor consistency between platforms, with correlation values ranging from a minimum of -0.267 for OAA vs. QiaSeq to a maximum of 0.407 for miRCURY vs. QiaSeq (**Supplementary Figure S4C**). The intersections between miRs with an area under ROC curve (AUC) value above or equal to 0.7 are shown with a Venn diagram in **Figure 5B**. Two ct-miRs, miR-150-5p and miR-210-3p, were in common to all the platforms. Although miR-210-3p upregulation in NSCLC was statistically significant only in QiaSeq (FDR <0.1), it displayed the same trend of modulation in the other platforms (data not shown). As shown in **Figure 5C** and **Supplementary Table S7**, the AUC value of miR-150-5p, including the single-assay qPCR results, ranged from 0.95 for QiaSeq [95% confidence interval (CI): 0.87–1] to 0.83 for miRCURY (95% CI: 0.64–1). The AUC for miR-210-3p ranged from 0.87 for OAC (95% CI: 0.7–1) to 0.71 for miRCURY (95% CI: 0.49–0.94). These results indicate that all platforms can detect the discriminatory power between NSCLC patients and healthy donors of these two ct-miRs, even if the accuracy is platform dependent.

### Validation of miR-150-5p and miR-210-3p as Potential Biomarkers in Tissues

**Figure 6:**
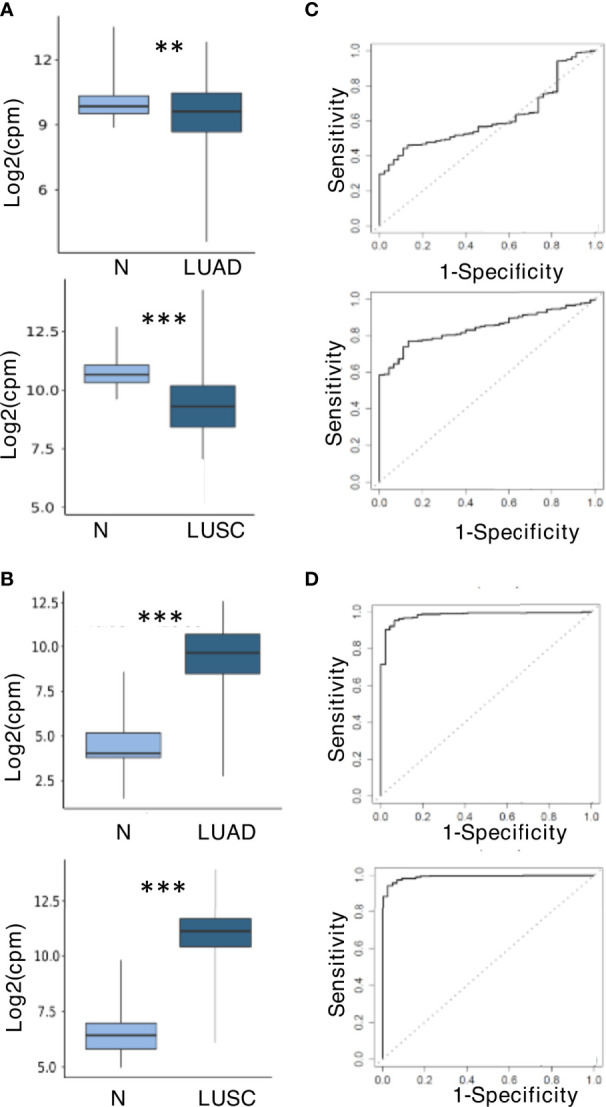
miR-150-5p and miR-210-3p expression and predictive value at tissue level in The Cancer Genome Atlas (TCGA) dataset. **(A)** Downregulation of miR-150-5p. **(B)** Upregulation of miR-210-3p. The upper and lower boxplots in **(A, B)** refer, respectively, to lung adenocarcinoma (LUAD) and lung squamous cell carcinoma (LUSC) compared with normal lung samples in the TCGA cohort. The log_2_ (fold change) values for the magnitude of difference are as follows: -0.56 (LUAD vs. normal) and -1.45 (LUSC vs. normal) for miR-150-5p; 5.02 (LUSC vs. normal) and 4.46 (LUSC vs. normal) for miR-210-3p. The *P*-value by unpaired two-tailed Student’s *t*-test are as follows: **P ≤ 0.01; ***P ≤ 0.001. **(C)** Receiver operating characteristic (ROC) curves for miR-150-5p. **(D)** ROC curves for miR-210-3p. LUADs are displayed in the upper boxplots, and LUSC are in the lower boxplots of **(C, D)**.

To further explore the role of miR-150-5p and miR-210-3p as potential biomarkers even for NSCLC tissues, the TCGA miR sequencing data for tumors and normal tissues of patients affected by LUAD and LUSC were analyzed. The results shown in **Figures 6A, B** indicated that the trend of dysregulation of these two miRs at the tissue level agreed to that observed in plasma. In comparison with normal tissues, the downregulation of miR-150-5p was however higher for LUSC than LUAD (**Figure 6A**). In contrast, miR-210-3p was significantly upregulated in both histologies (**Figure 6B**). ROC curve analysis was performed to evaluate the diagnostic value of the two miRs at the tissue level. As shown in **Figures 6C, D**, they appeared to represent valuable diagnostic markers. The miR-210-3p AUC values were high in both LUAD and LUSC cohorts at 0.98 and 0.99, respectively (**Figure 6D**), whereas those for miR-150-5p had higher AUC in LUSC (0.84) than in LUAD (0.61) (**Figure 6C**). The related data corresponding to AUCs are summarized in **Supplementary Table S7**.

## Discussion

We here analyzed the miR profiles of the plasma fluids of 10 NSCLC lung cancer patients and 10 healthy donors by using five different high-throughput platforms that are among the most commonly used commercially available technologies.

Each platform was assessed for performance parameters (intra-platform reproducibility, detection rate, and inter-platform correlation), for MSC classification concordance, and for the ability to detect differences between biological groups (*e*.*g*., healthy individuals and patients). The ct-miR detection rate was more similar across qPCR technologies. QiaSeq exhibited the highest miR counts in all sample groups, indicating that it is a true discovery technology that greatly expands miR repertoire detection and allows the identification of novel miRs. In contrast, EdgeSeq, directly performed on crude human plasma specimens without RNA extraction, displayed the lowest sensitivity. The intra-platform reliability, assessed by calculating pairwise concordance correlation coefficients between duplicates, was very high for all platforms except for OAA which had slightly lower CCC values. The results from the inter-platform reproducibility are consistent with those of previous studies, indicating that the overlap between different technologies is small (17–21). Our clustering analysis demonstrated that the correlation between ct-miRs depends on the platform and on the expression level of the miRs. The highest inter-platform reproducibility was observed between the qPCR-based platforms miRCURY, OAC, and OAA. EdgeSeq, which displayed a very high number of miRs with an expression value of 0 after background correction, had the lowest number of miRs, showing a low inter-platform correlation. It is the only technology that performs direct miR-targeted sequencing without RNA extraction procedures, and the results probably reflect the lower sensitivity for the quantification of low-abundance miRs as already reported (19). However, for specific highly expressed ct-miRs such those in cluster 4, it showed an inter-platform correlation comparable with the other platforms. Our study pinpoints the challenges inherent to the choice of a downstream detection technology for ct-miR profiling in a clinical setting and advises the use of a dual-platform approach to overcome the limitations of single platforms. If cost will prevent this approach, the aim of the experiment should be considered. At a discovery stage, unbiased high-throughput screens of miRs like that offered by QiaSeq small-RNA sequencing could be recommended. High-throughput qPCR technique by miRCURY or OAC could be also a good option for discovery as well as for more focused studies. Regardless of the platform used, putative biologically relevant miR biomarkers should be further validated by an independent technology. Except for EdgeSeq, concordance of MSC classification to the gold-standard assay was high for all other platforms, in particular, for OAA, miRCURY, and QiaSeq, establishing that the classifier could be reproducibly implemented in other multiplexed platforms.

Despite the fact that many studies investigating plasma miRs in patients with NSCLC provide evidence of the potential value of ct-miRs as non-invasive biomarkers, uncertainties remain regarding the clinical validity and utility of dysregulated ct-miRs for lung cancer diagnosis, prognosis, and prediction of response to treatment (35, 36). There are many reasons underlying the variability among published studies, including the use of different technologies and platforms, as also shown here, and heterogeneity of clinical cohorts. Indeed a recent multicentric study in the context of the EU network CANCER-ID reported low concordance among the miR results obtained by comparing two hybridizations (Toray 3D and nCounter), one sequencing (QiaSeq), and two qPCR (miRCURY and two-tailed qPCR) on biological samples composed of cell-free and extracellular-derived miR fractions from NSCLC patients (*n* = 27) and healthy control samples (*N* = 20) (20). In addition, no common DE ct-miRs among cancer patients and donors were detected by the different quantification technologies. This result could be related to the composition of the NSCLC cohort which included different stages of the disease before and during systemic treatment or radiotherapy as well as before and after surgery and to the control cohort not age-matched with that of the patients (20).

Though smaller, our NSCLC cohort was properly matched with healthy heavy smoker donors and allowed the detection of differential ct-miR expression between cancer patients and controls. The ability to detect statistically significant DE ct-miRs was platform dependent. However, when we disregarded the statistical significance and we focused on the direction of the modulation, we observed that most of the ct-miRs were concordant, except for EdgeSeq and OAA that displayed a high number of discordant DE ct-miRs. Finally, all platforms identified miR-150-5p and miR-210-3p as the best circulating biomarkers able to discriminate NSLC patients from healthy donors. Of note is the fact that since these two groups were matched for MSC test results, we can speculate that miR-150-5p and miR-210-3p are diagnostic markers independent of the MSC test result.

They were also confirmed at the tissue level, where the same trend of significant differences was observed in comparison with healthy tissue. miR-150-5p was also validated in the same plasma samples using single-assay qPCR, which is considered the gold-standard method for expression quantification.

miR-150-5p plays a critical role in the development of lymphoid and myeloid lineages in both mice and humans and has been observed to be dysregulated in solid and hematological malignancies where, depending on the context, it can exert concogenic or oncosuppressor functions (37, 38). Several lines of evidence point to its downregulation in different human cancers, like head and neck squamous carcinoma, cholangiocarcinoma, prostate, and hepatocellular carcinoma (39–42), supporting the tumor suppressor role of miR-150-5p. In addition, it was found downregulated in non-neoplastic diseases like advanced heart failure, critical illness, and sepsis (43, 44). The results on the expression and role of miR-150-5p in NSCLC are however conflicting since both oncogenic and tumor suppressor functions have been reported (37, 45–48). Its expression level, as detected in tissues by *in situ* hybridization, negatively correlates with metastasis, including lymph node and distant metastasis, at the time of diagnosis (45). The follow-up data indicate that patients with a low expression of miR-150-5p have a poor progression-free survival rate and a poor overall survival rate compared with those with high miR-150-5p expression (45). Conversely, as assessed by qPCR, the expression of miR-150-5p was found at levels significantly more elevated in NSCLC in comparison with that in non-tumor tissues (46). At the circulating level, once again, either up- and downregulation in plasma or serum of different cohorts of NSCLC patients in comparison with healthy donors was reported (49, 50). By profiling blood plasma miRs in NSCLC patients and healthy individuals using the miRCURY platform with the LNA qPCR Serum/Plasma Panel, the upregulation of miR-210 and the downregulation of miR-150-5p were observed for both pre-miR and mature miR levels (49). Our results agree with the above-described studies but contradict the finding indicating that the plasma levels of miR-150 and miR-210, among a panel of 12 candidate miRs, were both significantly upregulated in the plasma of NSCLC patients compared with healthy controls (50). As previously mentioned, several parameters like differences in research design, populations and specimens, and experimental methods can be relevant for inconsistencies from study to study. In addition, normalization of expression is a common challenge of miR studies in biological fluids in the absence of stable normalizers. Therefore, the function of miR-150-5p in NSCLC warrants further investigations. Nonetheless, it is worth to point out that, in our study, by applying distinct normalization strategies for data derived from small RNA sequencing, high-throughput qPCR methods, and individual qPCR assay, miR-150-5p was found to be coherently downregulated in plasma samples from NSCLC patients by five different miR profiling platforms, starting from different materials (RNA and crude plasma) and further validated by a single assay.

The role of miR-150-5p downregulation in the early diagnosis of lung tumor development is further supported by recent findings in chronic obstructive pulmonary disease, often associated with comorbidities and an increased risk of cancer, in a large-scale collection of samples from patients without cancer at baseline but with follow-up data (51–54). An increasing number of new strategies for therapeutic miR approaches are currently being pursued to restore the level of downregulated miRs and regain their tumor suppressor function (55). miR-150-5p activity as tumor suppressor has been related to its ability to inhibit wingless (Wnt)-β-catenin signaling pathway, closely associated with NSCLC progression, by targeting known activators like glycogen synthase kinase 3 beta interacting protein, β-catenin, and high mobility group AT-hook 2 (45, 56) as well as to reduce the matrix metalloproteinase 14 (MMP14) levels, whose overexpression correlates with a poor prognosis in NSCLC patients (47–57). The regulation of miR-150-5p is complex, and several long noncoding RNAs or circular RNAs can promote NSCLC cell growth and metastasis through sponging miR-150-5p (58–62).

At difference to miR-150-5p, miR-210-3p has been unambiguously described as a promising biomarker for NSCLC lung cancer due to its upregulation at the tissue, plasma, and serum levels and to its discriminatory accuracy in patients *versus* healthy controls (10, 12, 63–71). Investigations into the effects of miR-210 on lung cancer cell behavior as well as into the specific mechanisms underlying the role of miR-210 in the pathogenesis of NSCLC have been performed. It has been shown to regulate proliferation and apoptosis by targeting the transcriptional regulator SIN3A (69), a tumor suppressor gene for NSCLC cells (70). In addition, exosomal miR-210-3p derived by cancer stem cells targets fibroblast growth factor receptor-like 1 to elicit a pro-metastatic phenotype (71).

In conclusion, our study provides a comparison of ct-miRs, relevant in NSCLC, using widely used high-throughput platforms. We could show that the correlation between ct-miRs depends on both the type of platform and the miRs expression levels. Indeed a high inter-platform correlation was observed for ct-miRs profiled in qPCR-based platforms and, for all platforms, within highly expressed ct-miRs. Concordance of MSC classification among most miR detection technologies with the “gold-standard” method established that the classifier could be successfully implemented in other multiplex platforms. Finally, we here demonstrate, for the first time, that the decreased abundance of miR-150-5p and the increased abundance of miR-210-3p in the plasma of lung cancer patients is independent of the detection technology. Both miRs display promising attributes and constitute attractive circulating biomarkers for NSCLC cancer detection. Larger and prospective studies composed of patients with different NSCLC histological cancer subtypes and at different stages of the disease are needed to confirm their significance.

## Data Availability Statement

The datasets presented in this study can be found in online repositories. The names of the repository/repositories and accession number(s) can be found below: https://www.ncbi.nlm.nih.gov/geo/, GSE204951. Normalized data are available in **Supplementary Table S8**.

## Ethics Statement

Ethical review and approval were not required for the study on human participants in accordance with the local legislation and institutional requirements. The patients/participants provided their written informed consent to participate in this study.

## Author Contributions

MS, LDC, GS, MB, and MD conceived and designed the study. MB and GS provided samples. LDC, AM, MFI, MB, AM, and EM analyzed the cases. CG, MD, and MB conducted the computational analyses. MS, MD, and CG contributed to the preparation of the original draft. All authors contributed to the article and approved the submitted version

## Funding

This research was supported by grant 12162 (Special Program “Innovative Tools for Cancer Risk Assessment and Early Diagnosis” 5 × 1000) from the Italian Association for Cancer Research (to GS as PI and MS as GL) and by 5 × 1000 Funds (Italian Ministry of Health 2014—institutional grant BRI2017) from Fondazione IRCCS Istituto Nazionale dei Tumori (to LDC as PI) and by Italian Ministry of Health (Ricerca Corrente 2022 Funds).

## Conflict of Interest

GS and MB are coinventors for three patent applications licensed to Gensignia Life Sciences and regarding the miR MSC signature used in this article.

The remaining authors declare that the research was conducted in the absence of any commercial or financial relationships that could be construed as a potential conflict of interest.

## Publisher’s Note

All claims expressed in this article are solely those of the authors and do not necessarily represent those of their affiliated organizations, or those of the publisher, the editors and the reviewers. Any product that may be evaluated in this article, or claim that may be made by its manufacturer, is not guaranteed or endorsed by the publisher.

## Supplementary Material

Supplementary Figure 1
**(A)** Venn diagram reporting the intersection of the miRs present on all platforms, highlighting the 488 in common. **(B)** Boxplots representing the number of detected ct-miRs in each platform with respect to those present in each platform. **(C)** Boxplots reporting the percentage of GC in detected (pink) and undetected (blue) ct-miRs for each platform. *P*-value calculation by Wilcoxon rank-sum test. **(D)** Comparison of the empirical cumulative density function for each platform of the expression quantiles of the 26 ct-miRs detected in all samples and in all five platforms.

Supplementary Figure 2 Scatterplot of duplicate profiles derived from three plasma samples (#1, NSCLC patient; #2 and #3, donors) calculated on normalized and filtered data in each platform. The platforms from top to bottom are OAC, OAA, miRCURY, EdgeSeq, and QiaSeq.

Supplementary Figure 3 Comparison of differential expression results across platforms. **(A)** Number of DE ct-miRs in each platform according to different false discovery rate (FDR) thresholds. **(B)** Number of DE ct-miRs shared by at least 2, 3, 4, and 5 platforms according to different FDR thresholds. **(C)** Pairwise scatter plots of the *t*-statistic values obtained from the differential expression analysis performed for each platform. Pearson’s correlation coefficients and significance are reported. ****p*-value is <0.001, ***p*-value is <0.01, and **p*-value is <0.05.

Supplementary Figure 4 Comparison of area under the curve (AUC) values of ct-miRs across platforms. **(A)** Number of ct-miRs in each platform with AUC values above increasing cutoffs. **(B)** Number of ct-miRs shared by at least 2, 3, 4, and 5 platforms according to increasing AUC thresholds. **(C)** Pairwise scatter plots of AUC values for the classification of lung cancer patients and healthy donors. Pearson’s correlation coefficients and significance are reported. ****p*-value is <0.001, ***p*-value is <0.01, and **p*-value is <0.05.
