# Supplementary figures and images for: A Cross-Comparison of High-Throughput Platforms for Circulating MicroRNA Quantification, Agreement in Risk Classification, and Biomarker Discovery in Non-Small Cell Lung Cancer
Source: Front Oncol. 2022 Jul 19;12:911613. doi: 10.3389/fonc.2022.911613 (PMC9343840; doi:10.3389/fonc.2022.911613)

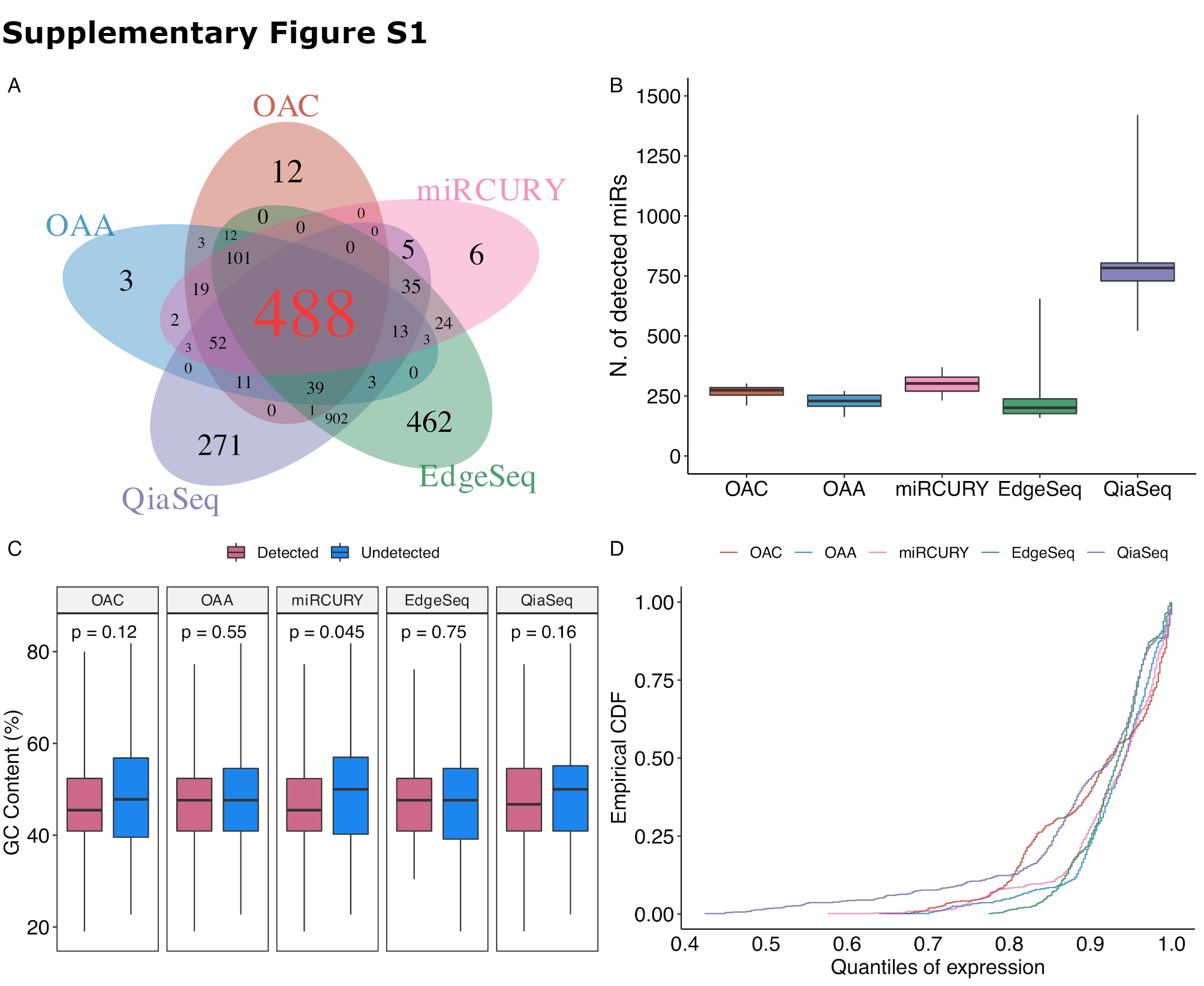

Supplement: Supplementary Figure 1 — (A) Venn diagram reporting the intersection of the miRs present on all platforms, highlighting the 488 in common. (B) Boxplots representing the number of detected ct-miRs in each platform with respect to those present in each platform. (C) Boxplots reporting the percentage of GC in detected (pink) and undetected (blue) ct-miRs for each platform. P-value calculation by Wilcoxon rank-sum test. (D) Comparison of the empirical cumulative density function for each platform of the expression quantiles of the 26 ct-miRs detected in all samples and in all five platforms. [file Image_1.tif]

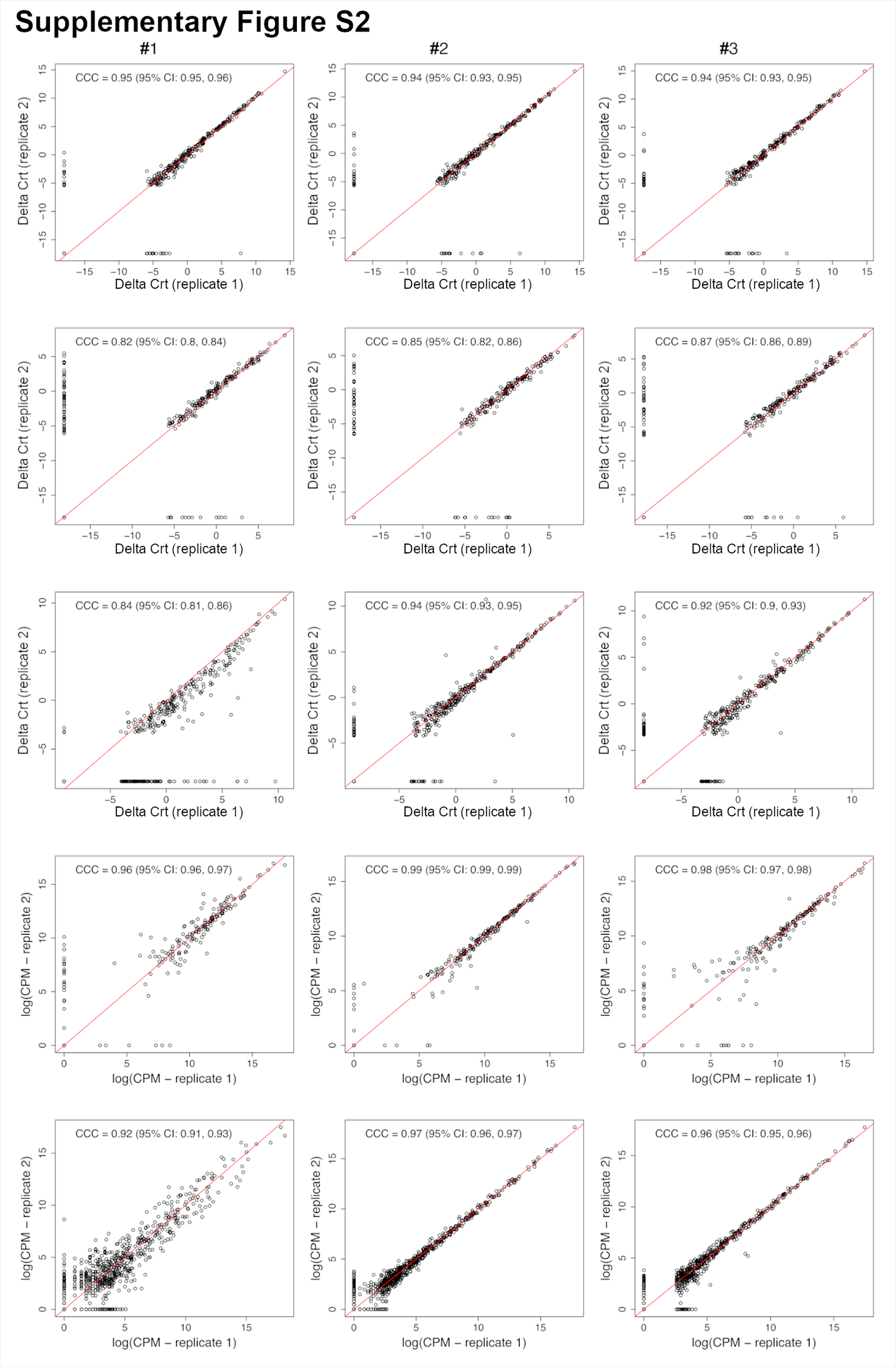

Supplement: Supplementary Figure 2 — Scatterplot of duplicate profiles derived from three plasma samples (#1, NSCLC patient; #2 and #3, donors) calculated on normalized and filtered data in each platform. The platforms from top to bottom are OAC, OAA, miRCURY, EdgeSeq, and QiaSeq. [file Image_2.tif]

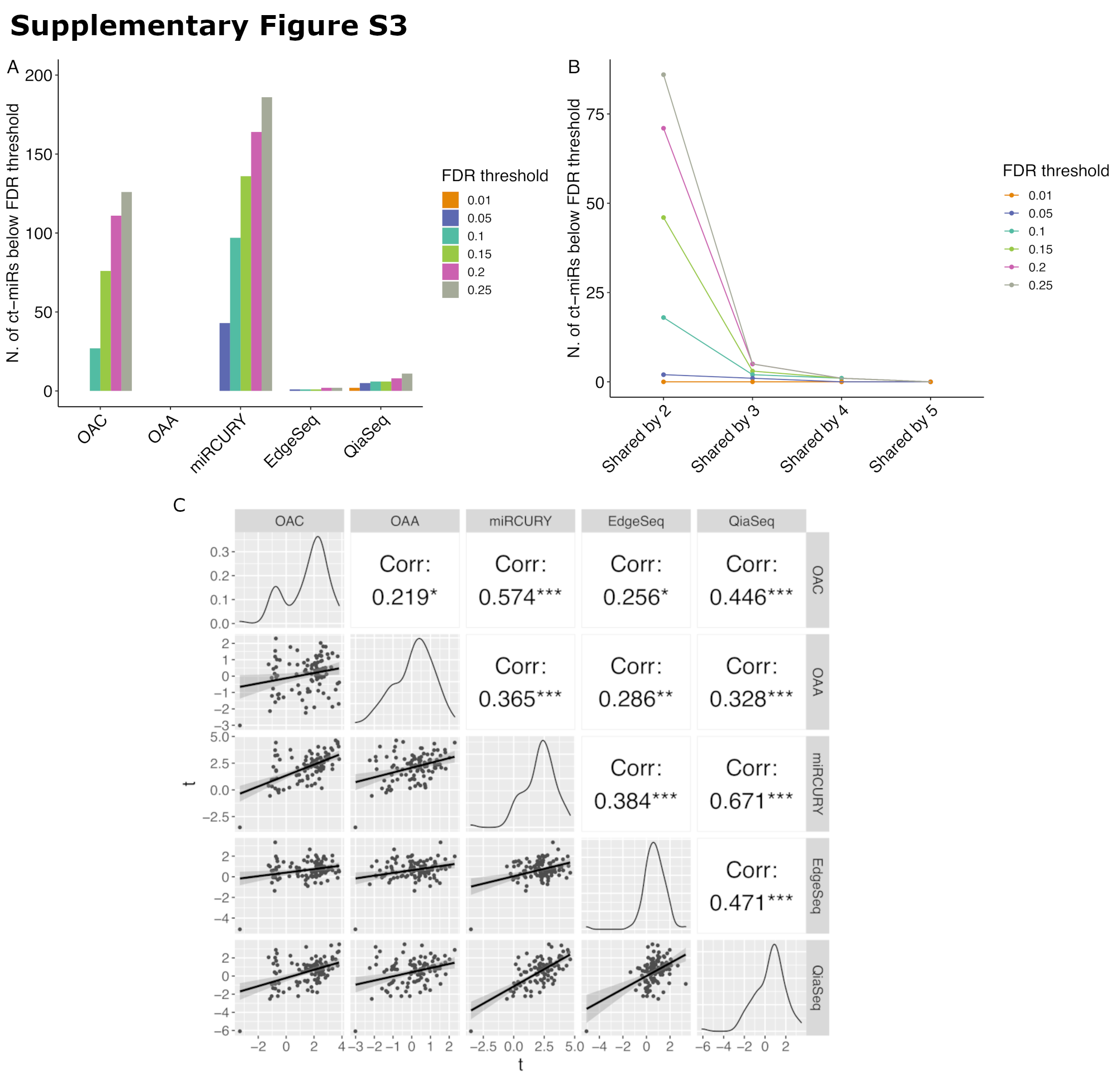

Supplement: Supplementary Figure 3 — Comparison of differential expression results across platforms. (A) Number of DE ct-miRs in each platform according to different false discovery rate (FDR) thresholds. (B) Number of DE ct-miRs shared by at least 2, 3, 4, and 5 platforms according to different FDR thresholds. (C) Pairwise scatter plots of the t-statistic values obtained from the differential expression analysis performed for each platform. Pearson’s correlation coefficients and significance are reported. ***p-value is <0.001, **p-value is <0.01, and *p-value is <0.05. [file Image_3.tif]

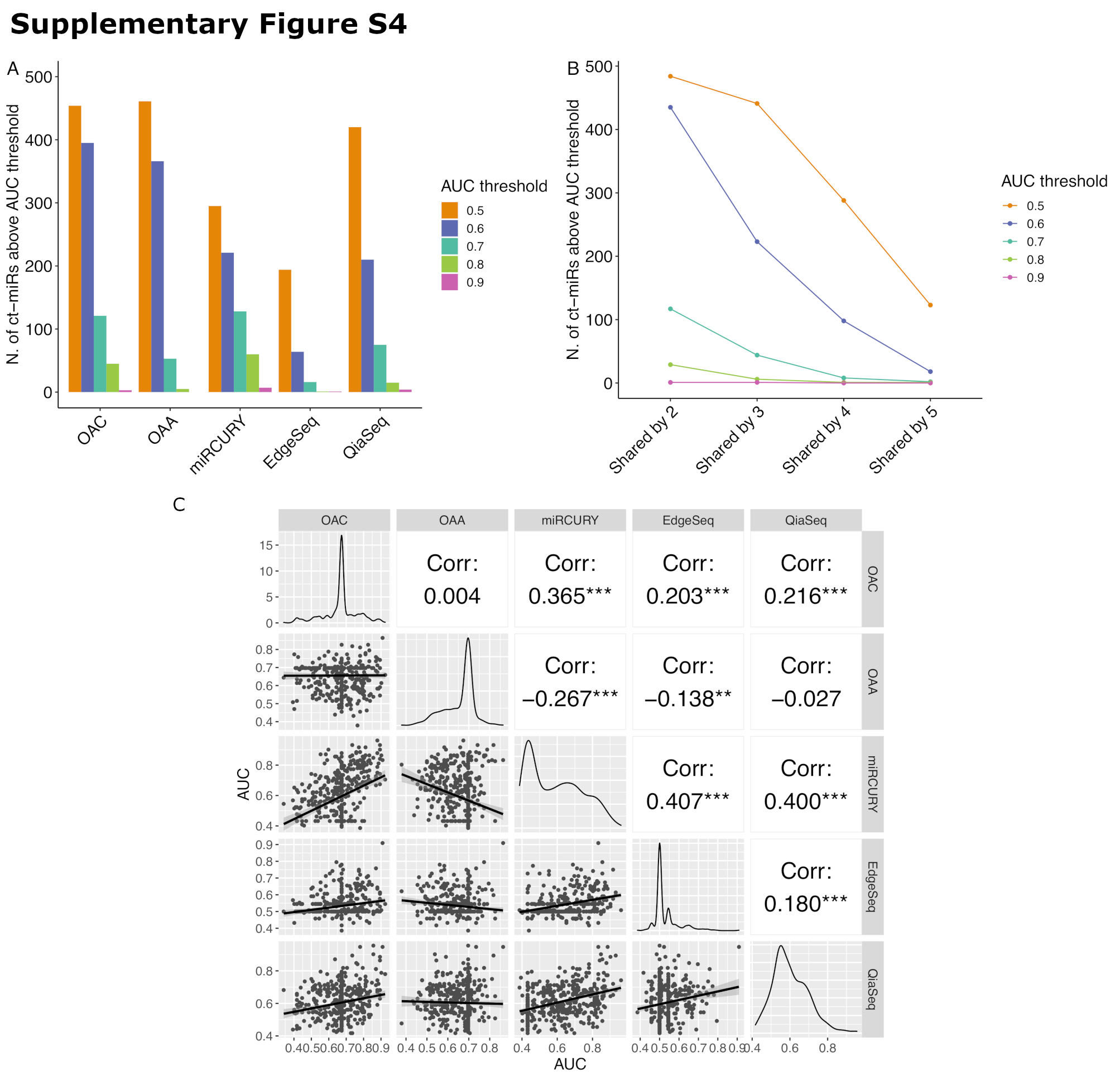

Supplement: Supplementary Figure 4 — Comparison of area under the curve (AUC) values of ct-miRs across platforms. (A) Number of ct-miRs in each platform with AUC values above increasing cutoffs. (B) Number of ct-miRs shared by at least 2, 3, 4, and 5 platforms according to increasing AUC thresholds. (C) Pairwise scatter plots of AUC values for the classification of lung cancer patients and healthy donors. Pearson’s correlation coefficients and significance are reported. ***p-value is <0.001, **p-value is <0.01, and *p-value is <0.05. [file Image_4.tif]
